# A novel pyroptosis risk model composed of *NLRP6* effectively predicts the prognosis of hepatocellular carcinoma patients

**DOI:** 10.1002/cam4.4898

**Published:** 2022-06-01

**Authors:** Xin Gao, Wen‐Xin Wang, Xiao‐Lan Zhang

**Affiliations:** ^1^ Department of Gastroenterology The Second Hospital of Hebei Medical University Shijiazhuang Hebei Province China

**Keywords:** hepatocellular carcinoma, immune alterations, molecular classification, *NLRP6*, prognostic signature, pyroptosis landscape, tumor microenvironment

## Abstract

**Background:**

Pyroptosis is a unique inflammatory‐related cell death process, and inflammation is considered to be a key factor in hepatocellular carcinoma (HCC). However, the pyroptosis landscape in HCC has not been thoroughly studied.

**Methods:**

Clinical data and RNA sequencing data of HCC patients were collected from The Cancer Genome Atlas database, and differentially expressed genes (DEGs) associated with pyroptosis were discovered. The relationship between DEGs and prognosis was studied. Using The Cancer Genome Atlas cohort, a least absolute shrinkage and selection operator regression model was built on the basis of pyroptosis‐related DEGs, which was then verified by the Gene Expression Omnibus (GEO) cohort. Functional enrichment analysis and immunological states were also studied between distinct risk subgroups. Finally, the potential tumor suppressive function of *NLRP6* in HCC was analyzed.

**Results:**

In total, 26 pyroptosis‐related DEGs were identified. Consensus clustering results showed patients with high levels of pyroptosis were associated with higher tumor stage, grade, and poor prognosis. The least absolute shrinkage and selection operator risk model was built using six genes linked with prognosis (*GSDMC, GSDME, NOD2, NLRP6, CASP8,* and *SCAF11*). Risk score was an independent risk factor that suggested shortened overall survival in both the training cohort (HR: 3.52, 95% CI: 1.351–9.193) and validation cohort (HR: 3.31, 95% CI: 1.435–7.617). Meanwhile, the low‐risk population had higher immunological activity. We also found a novel potential tumor suppressor gene *NLRP6,* which may negatively regulate the E2F and MYC pathways and be associated with higher immune cell infiltration levels, which lead to better prognosis.

**Conclusions:**

This study revealed the pyroptosis landscape of HCC and provided a promising clinical prognosis evaluation model. Additionally, some new targets related to prognosis such as *NLRP6* are proposed for further study.

## Graphical abstract

In this study, we constructed a risk model using six genes linked with prognosis, which can accurately predict the overall survival of patients with hepatocellular carcinoma in both the training cohort and the validation cohort. Further, we highlighted that NLRP6 is a novel protective gene, may negatively regulate E2F and MYC pathways, resulting in better prognosis.

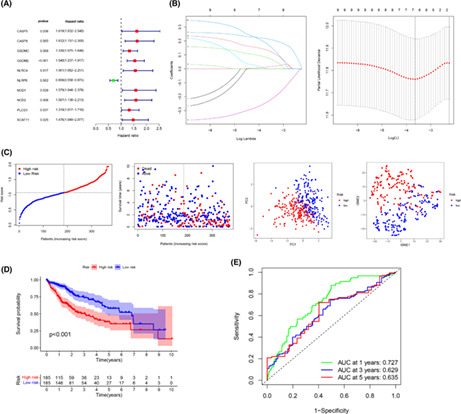

## 1 INTRODUCTION

Hepatocellular carcinoma (HCC) is often secondary to chronic hepatitis and is one of the most lethal malignant tumors in humans. Annually, approximately 906,000 cases of HCC are diagnosed worldwide,^1^ and in China, the 5‐year overall survival (OS) rate for HCC is less than 15%.^2^, ^3^ Some new treatment strategies and chemotherapy medicines have been proposed^4^; however, there remains a need to find new driver molecules that can serve as biomarkers to predict prognosis or therapeutic targets to improve the prognosis of HCC patients.

Molecular typing using genomic differences is a promising strategy that reflects the inter‐tumor heterogeneity of biological functions that lead to the differences in prognosis.^5^ Some prognostic models for HCC have been proposed, such as using immune characteristics,^6^ ferroptosis,^7^ and autophagy.^8^ Inflammation plays an important role in the occurrence and treatment of HCC. Therefore, mechanisms related to inflammation may provide a new perspective for classifying HCC.

Pyroptosis is a newly discovered programmed death process that involves the Gasdermin family and is frequently triggered by inflammatory caspase activation.^9^, ^10^, ^11^ Recently, there has been increased interest in the mechanisms of pyroptosis in tumor growth and invasion, and pyroptosis‐related gene (PRG) signatures have been described in multiple human cancer types, such as gastric cancer and colon cancer.^12^, ^13^, ^14^, ^15^, ^16^, ^17^ In liver diseases, the IL‐1β and IL‐18 secreted by cells undergoing pyroptosis can induce inflammatory responses and cause liver damage, which is closely related to hepatitis and cirrhosis.^18^ In HCC, caspase‐1 inhibitors can reduce the proliferation, migration, and invasion of HCC cells, but under hypoxic conditions, caspase‐1 activation promotes the release of inflammatory factors that accelerate HCC cells achieving epithelial‐mesenchymal transition; thus, enhancing their invasive and metastatic abilities.^19^, ^20^ However, to date, only a limited number of prognostic PRGs have been identified for HCC. This study systematically reviewed data associated with PRG expression and clinical information in HCC patients and established a promising risk model to predict the outcomes of HCC patients. Moreover, some new driver molecules such as *NLRP6* are proposed for the first time, which is helpful to improve the clinical diagnosis and treatment for HCC patients.

## 2 MATERIALS AND METHODS

### 2.1 Datasets

RNA sequencing transcriptome profiling and clinical data of 374 liver HCC samples and 50 normal control samples were obtained from The Cancer Genome Atlas (TCGA) database (Liver Hepatocellular Carcinoma [LIHC] cohort). Four HCC patients were excluded because of a lack of complete follow‐up information in the survival analysis. The corresponding data were obtained from the Gene Expression Omnibus (GEO) database for further verification, including 78 HCC patients (ID: GSE54236^21^). We used a panel of 33 pyroptosis‐related genes that has been reported by previous studies.^22^, ^23^ Table S1 is a comparison of the clinicopathological characteristics between the training cohort and testing cohort, and Figure S1 shows the flow chart for this study.

### 2.2 Identification of PRGs

The “limma” R package was used to identify pyroptosis‐related differentially expressed genes (DEGs) between tumor and normal tissues in TCGA cohort. Interactions between DEGs were investigated using a protein–protein interaction (PPI) network and the “igraph” R package. We use the Gepia platform^24^ to run a Kaplan–Meier (K–M) survival analysis to see if there was a link between prognosis and each DEG (*p* < 0.05). GSCAlite^25^ was also used to analyze the mutation mode of PRGs.

### 2.3 Consensus clustering

The “ConsensusClusterPlus” R program was used to divide TCGA‐LIHC patients into two subgroups based on the DEGs found.^26^

### 2.4 Construction and validation of the least absolute shrinkage and selection operator (LASSO) regression model

We performed Cox regression analysis using “survival” R packages to further identify the prognostic values of pyroptosis‐related DEGs. The LASSO risk model was created using “GLMnet” R package, and 10× cross‐validation was carried out,^27^ which included six genes (*GSDMC, GSDME, NOD2, NLRP6, CASP8,* and *SCAF11)*. We used “scale” function in R for centralization and standardization of the gene expression of TCGA‐LIHC, and risk scores were then calculated using the following formula: Risk Score = ∑^6^_i_X_i_ × Y_i_, where X is the risk coefficients and Y is gene expression level. The GEO validation cohort was standardized by “sva” R package to calculate the risk score of each patient. Finally, we systematically evaluated the subgroup distribution and prognostic value of the risk model in both cohorts, which included riskplot (“pheatmap” R packages), OS K‐M curves (“survival” and “survminer” R packages), receiver operating characteristic (“survivalROC” R package), principal component analysis (PCA) and t‐distributed stochastic neighbor embedding (“tsne” R packages), and independent prognostic analysis (“survival” R packages).

### 2.5 Analysis of functional enrichment

The Gene Ontology (GO) and Kyoto Encyclopedia of Genes and Genomes (KEGG) enrichment studies were performed in “clusterProfiler” R package.

### 2.6 Evaluation of PRG‐associated immune status between different patterns

To further evaluate the immune status in two subgroups, we used “limma” R package to determine overall DEGs between the high‐ and low‐risk subgroups (|log2FC| ≥ 1 and FDR <0.05). Next, we used Single‐sample Gene Set Enrichment Analysis (ssGSEA) in the “GSVA” R package^28^ and immune cell infiltration analysis in the “CIBERSORT” R package^29^ to conduct an overall evaluation of PRG‐associated immune status and compare different patterns.

### 2.7 Relationship between *NLRP6* and clinicopathological parameters

According to the median expression of *NLRP6*, the patients were divided into the high and low expression groups. The differences in clinicopathological parameters between different groups were analyzed by the chi‐square test. Univariate and multivariate analyses were used to determine the independent prognostic value of *NLRP6*.

### 2.8 Functional enrichment analysis and immune infiltration evaluation related to *NLRP6*

We used “clusterProfiler” R package to perform Gene Set Enrichment Analysis (GSEA) on the basis of h.all.V7.2.symbols.gmt [Hallmarks] and corrected by the BH method. Finally, we used ssGSEA (“GSVA” R package) and Spearman correlation analysis to explore the relationship between *NLRP6* expression and immune infiltrating cells.

### 2.9 Statistical analysis

Statistical analyses and visualizations were performed with R software (version 4.0.3). Results were determined to be statistically significant at *p* < 0.05 (*p < 0.05, **p < 0.01, and ***p < 0.001).

## 3 RESULTS

### 3.1 Identification of pyroptosis‐related DEGs in TCGA‐LIHC

**FIGURE 1:**
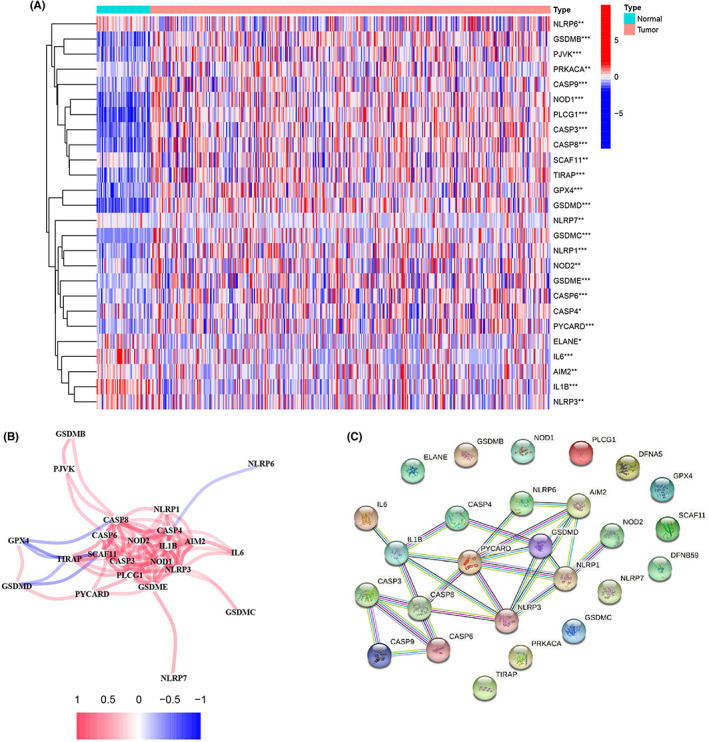
Expression of pyroptosis‐associated differentially‐expressed genes (DEGs) and interactions in the Liver Hepatocellular Carcinoma (LIHC) cohort. (A) heatmap of pyroptosis‐related gene expression in normal samples (N, blue) and tumor samples (green: Low expression level; red: high expression level) (T, red). (B) On the basis of a protein–protein interaction network (interaction score = 0.9), interactions of the 26 pyroptosis‐related DEGs. (C) The pyroptosis‐related DEGs correlation network (red line: Positive correlation; blue line: Negative correlation). The strength of the significance is reflected in the color depth

In 370 tumors and 50 normal samples, we investigated the expression levels of 33 pyroptosis‐related DEGs and found 26 DEGs. In tumor tissue, 23 genes were discovered to be upregulated, whereas three were found to be downregulated. Heatmaps of these mRNA levels are presented in Figure 1A. Meanwhile, correlations between pyroptotic‐related DEGs and the PPI network with the minimum needed interaction score (highest confidence 0.9) are shown in Figure 1B and C, respectively.

### 3.2 The relationship between DEGs linked to pyroptosis and prognosis

**FIGURE 2:**
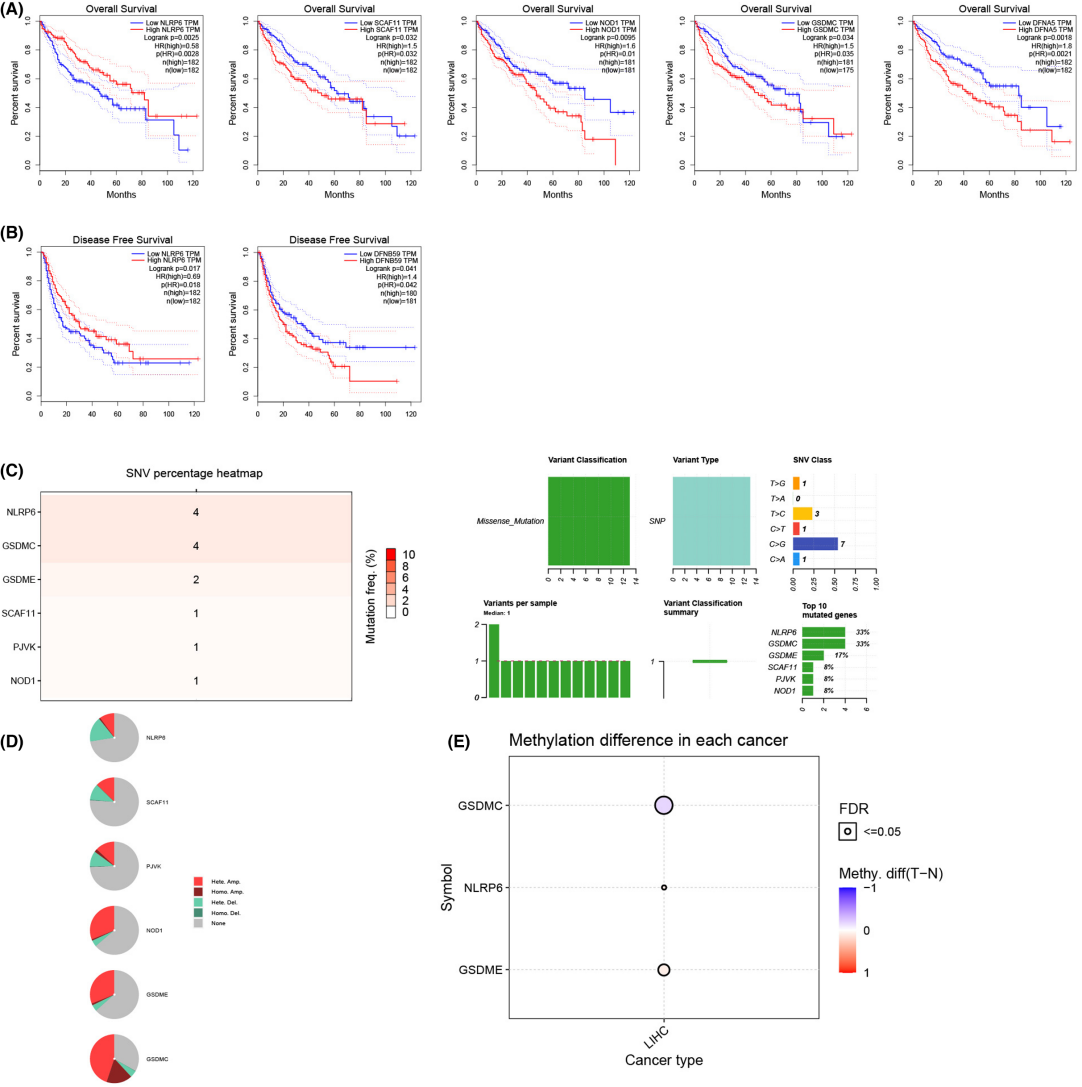
Prognostic value and mutation pattern of pyroptosis‐related DEGs. (A) Five genes were found to be associated with overall survival according to the Gepia platform (*DFNA5* and *GSDME*). (B) Two genes were associated with disease‐free survival (*DFNB59: PJVK*). (C) Single nucleotide variation landscape. (D) Copy number variation landscape. € Methylation status

We analyzed the association between the expression levels of 26 pyroptosis‐related DEGs and prognosis using the Gepia platform. Among them, four genes (*GMDSC, NOD1, SCAF11,* and *GSDME*) were harmful, while high expression of *NLRP6* was associated with better OS (Figure 2A). According to disease‐free survival data, we observed that high *NLRP6* expression and low *PVJK* expression were associated with better outcomes (Figure 2B). Subsequently, we investigated the types of mutations in genes associated with prognosis. The number of deleterious mutations in each gene is shown in Figure 2C, and C > G missense mutation was the main mutation mode. *NLRP6* showed more abundant heterozygous deletion mutations and *GSDMC* showed increased homozygous ploidy (Figure 2D). *NLRP6* and *GSDME* showed elevated methylation levels in tumor samples, while *GSDMC* showed the opposite characteristics (Figure 2E).

### 3.3 Consensus clustering on the basis of pyroptosis‐related DEGs

**FIGURE 3:**
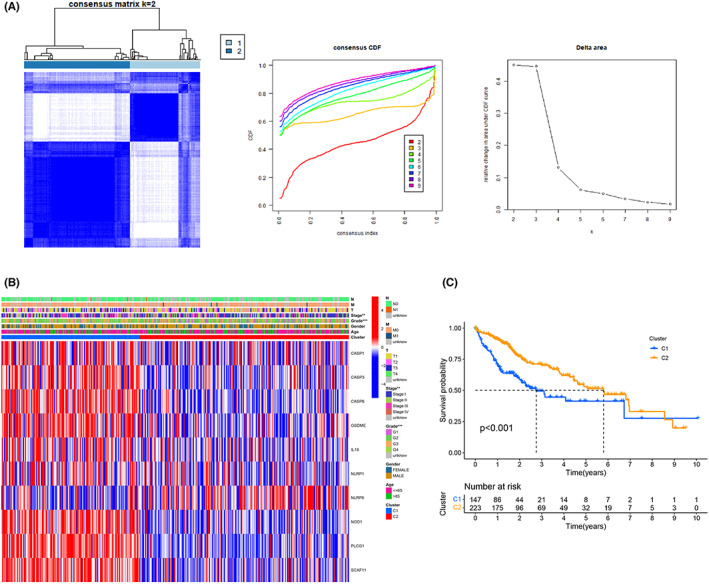
Consensus clustering according to DEGs associated with pyroptosis. (A) According to the consensus clustering matrix (k = 2), the 370 HCC patients were classified into two subgroups. (B) The heatmap shows the clinicopathological parameters and differential pyroptosis genes of the two patient clusters. (C) The prognosis of cluster1 patients was significantly better than that of cluster2

Consensus clustering analysis was performed for the TCGA‐LIHC cohort on the basis of the 26 previously obtained DEGs from the “ConsensusClusterPlus” R package to explore connections between these DEGs and LIHC subtypes. By increasing the clustering variable from 2 to 9, we found that the clustering stability was optimal when k = 2 (Figure 3A). Cluster2 (*n* = 223) had a better grade, stage, and prognosis than Cluster1 (*n* = 147) (*p* < 0.001, Figure 3B and C). Meanwhile, *IL18, GSDME, PLCG1, NOD1, CASP8, SCAF11, CASP1, NLRP1, CASP3,* and *NLRP6* were identified to be DEGs between the two subgroups (*p* < 0.05, Figure 3B). Molecular typing on the basis of pyroptosis‐related DEGs showed that elevated pyroptosis was a risk factor for HCC patients.

### 3.4 Construction of the LASSO regression model

**FIGURE 4:**
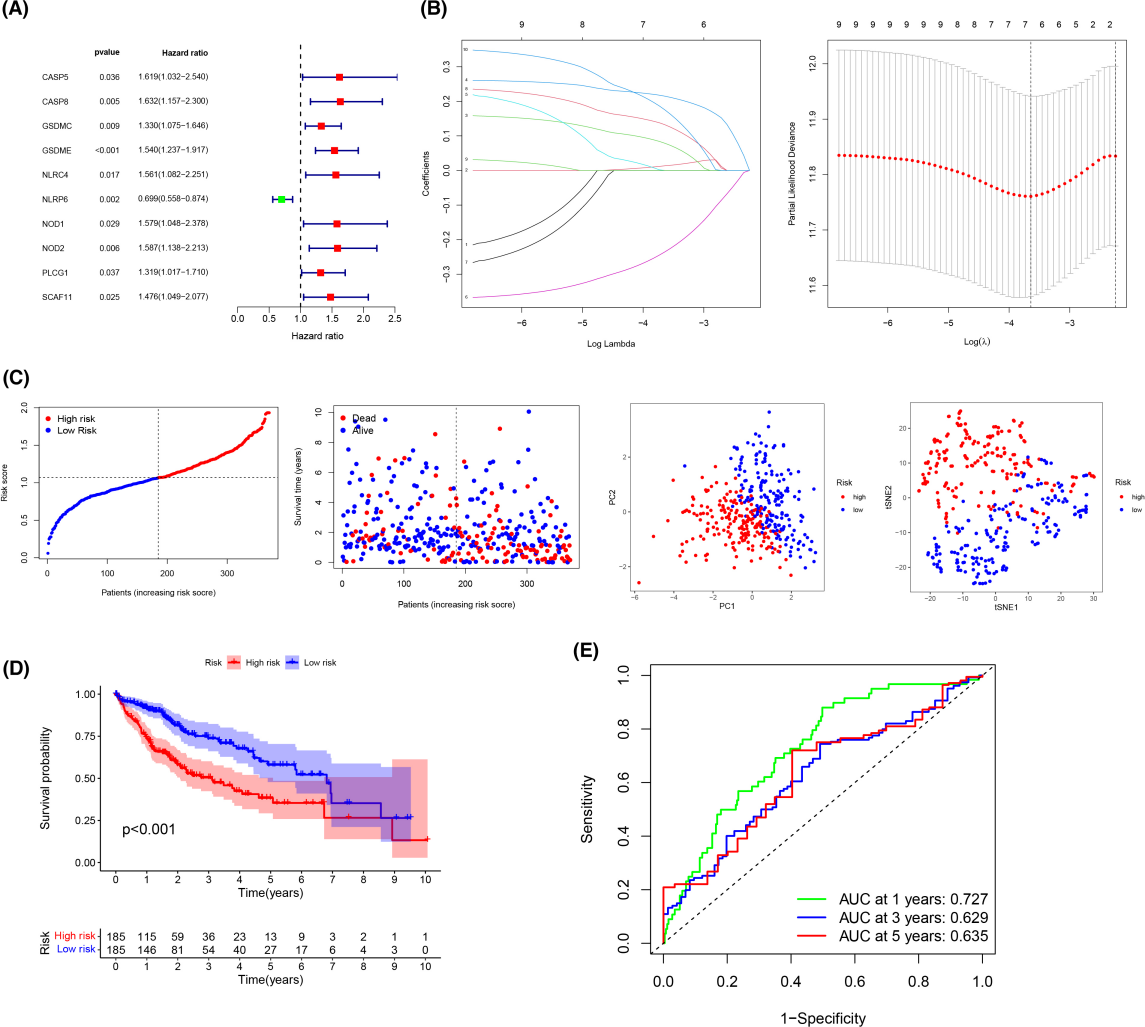
TCGA‐LIHC risk signature construction. (A) 10 DEGs with p < 0.05 according to univariate analysis. (B) Six genes were used to establish the LASSO regression model through cross‐validation. (C) Patients in the training cohort were well distributed according to their risk scores. (D) Kaplan–Meier curves for OS between the high‐risk group and low‐risk group in the training cohort. (E) Analysis of time‐dependent ROC scores

We used univariate cox regression analysis to further determine the prognosis‐related DEGs. Ten genes (*CASP5, CASP8, GSDMC, GSDME, NLRC4, NLRP6, NOD1, NOD2, PLCG1,* and *SCAF11*) were kept for further analysis; only one gene (*NLRP6)* was protective (Figure 4A). Six of the ten genes were chosen for LASSO Cox regression analysis to develop an optimal risk signature; the panel with six genes was the best solution (Figure 4B). The following formula was used to determine risk scores:

risk score = (*CASP8* exp. ×10.0134952699440289) + (*GSDMC* exp. × 0.0787539413788865) + (*GSDME* exp. × 0.214879318148402) + (*NLRP6* exp. × 0.235414131360918) + (*NOD2* exp. × 0.132326084157063) + (*SCAF11* exp. × 0.175276012924924).

On the basis of median risk score, the 370 TCGA‐LIHC patients were separated into two subgroups (Figure 4C). The dot plot depicts the survival status of the low‐ and high‐risk groupings, with the low‐risk population on the left side of the dotted line and the high‐risk group on the right side. Patients with varying risks were well segregated into the two clusters according to the PCA and *t*‐SNE results. The difference in OS between the two groups was substantial (*p* < 0.001, Figure 4D). The areas under the curves (AUCs) for 1‐year, 3‐year, and 5‐year survival were 0.727, 0.629, and 0.635, respectively (Figure 4E).

### 3.5 Validation in the GEO cohort

**FIGURE 5:**
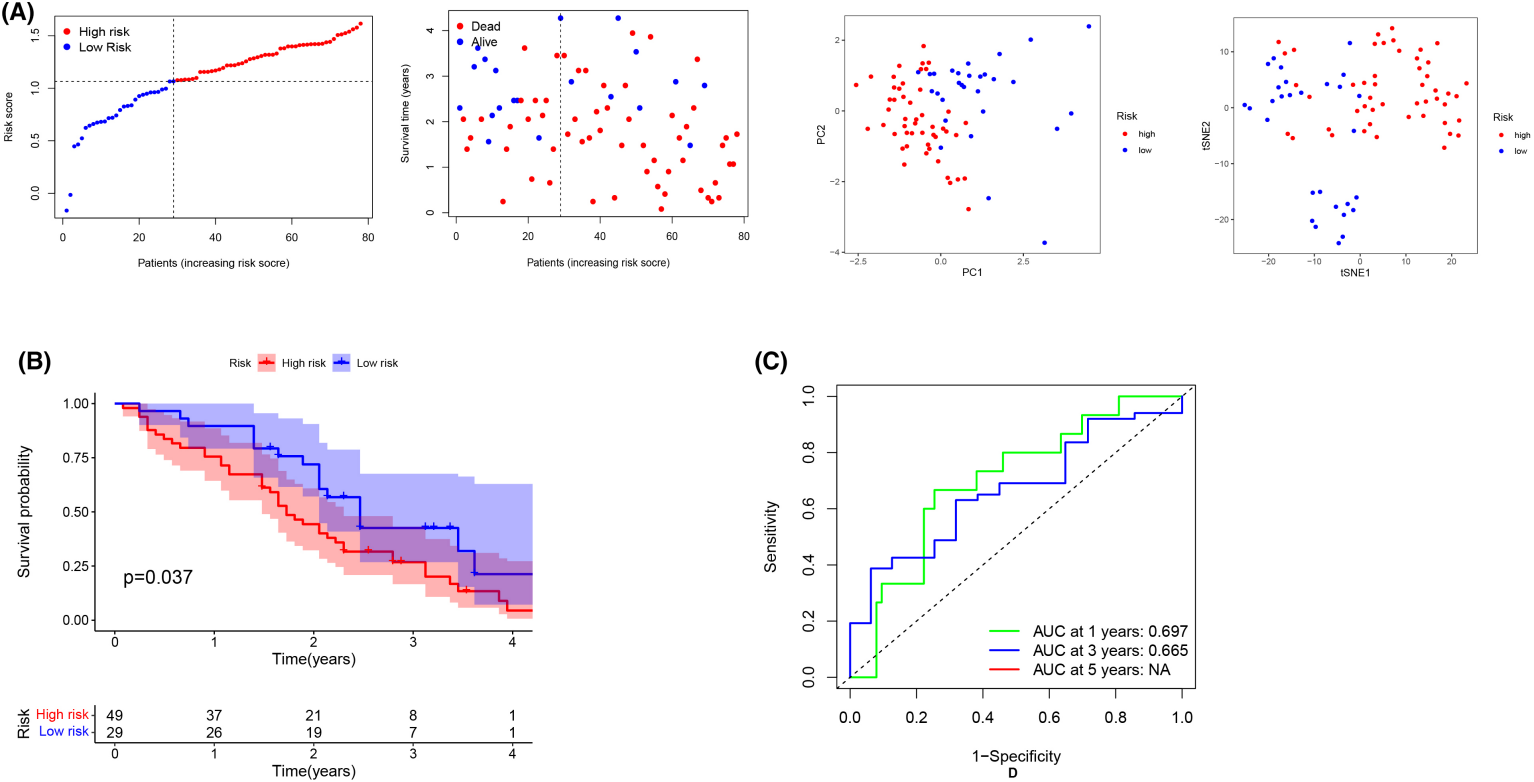
The LASSO risk model was validated by the Gene Expression Omnibus cohort. (A) Distribution according to risk scores. (B) Kaplan–Meier curves for OS in the validation cohort. (C) Analysis of time‐dependent ROC data

The risk signature's predictive power was next tested using an external cohort (GSE54236, *n* = 78). We used “Scale” function to normalize gene expression data of the test cohort according to the TCGA cohort. Risk ratings, which were produced using the same formula as bef ore, were used to classify patients into distinct subgroups, and patients in the high‐risk (*n* = 49) and low‐risk (*n* = 29) subgroups were well divided into two clusters using PCA and *t*‐SNE (Figure 5A). The K–M survival curves revealed a significant difference in OS between the two groups (*p* = 0.037) (Figure 5B). The AUC for 1‐year and 3‐year survival were 0.697 and 0.665, respectively. (Figure 5C). In conclusion, our results show that the pyroptosis‐related risk model composed of six genes can well predict the OS of patients with HCC, whether in TCGA cohort or test cohort.

### 3.6 Independent evaluation of the risk model's prognostic value

**FIGURE 6:**
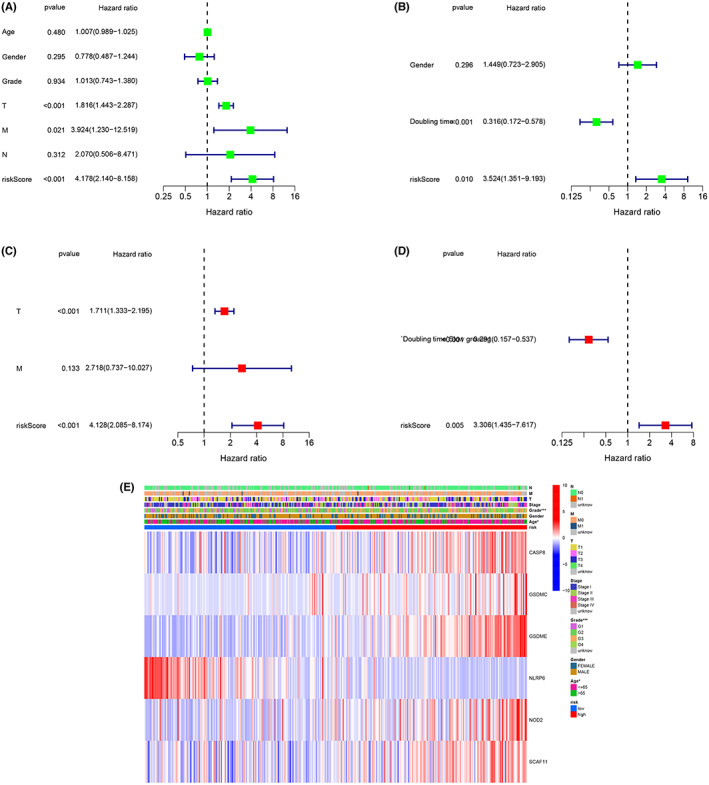
Independent prognostic value assessment of risk scores. (A) training and (B) validation cohorts by univariate analysis. (C) training and (D) validation cohorts by multivariate analysis. € Gene heatmap and clinical features of different risk subgroups according to the training cohort

To evaluate the risk model's independent prognostic efficacy, we conducted univariate and multivariate Cox regression analyses. In both the training and testing populations, high‐risk scores predicted poor survival (hazard ratio [HR]: 4.178, 95% confidence interval [CI]: 2.140–8.158; HR: 3.52, 95% CI: 1.351–9.193, respectively; Figure 6A and B). The risk score was also revealed to be an independent prognostic predictor for HCC patients after correcting for other confounding factors (HR: 4.128, 95% CI: 2.085–8.174; HR: 3.31, 95% CI: 1.435–7.617, respectively; Figure 6C and D). We also created a heatmap of clinical variables for TCGA cohort (Figure 6E) and discovered that age and tumor grade were distributed differently across the two categories. Combined with the results of the previous classification, we propose that the level of pyroptosis is most closely related to tumor grade, which may be a dynamic interaction.

### 3.7 Enrichment analysis of signaling pathways and evaluation of the tumor immune microenvironment

**FIGURE 7:**
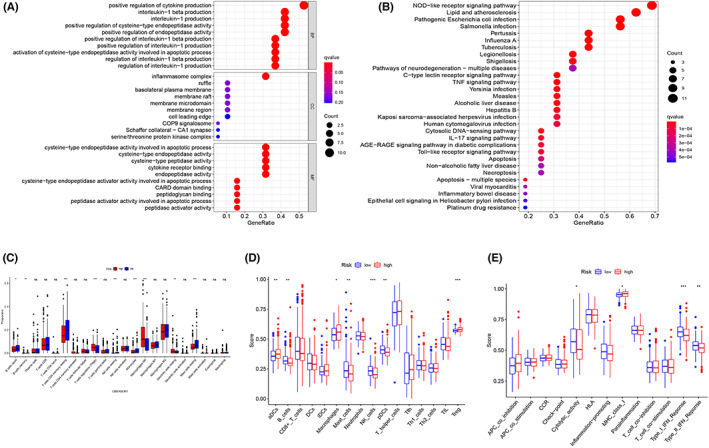
Functional enrichment analysis and evaluation of tumor immune infiltration. (A) Gene Ontology and (B) Kyoto Encyclopedia of Genes and Genomes analyses associated with the LASSO risk model (the number of genes enriched is represented by the size of the circles). (C) Levels of infiltrating immune cells according to CIBERSORT. (D) The immune cell scores and (E) and immune function evaluation according to ssGSEA

We used ssGSEA to further explore biological functions according to the risk score between the two subgroups. In GO analysis, DEGs were predominantly involved in cytokine production, interleukin‐1β generation, interleukin‐1 production, positive regulation of cycteine‐ type endopeptidase activity, inflammasome complex regulation, and other processes (Figure 7A). KEGG pathway analysis suggested that the NOD‐like receptor signaling pathway, Hepatitis B, pathogenic *Escherichia coli* infection, *Salmonella* infection, and the TNF signaling pathway were all involved in the DEGs, among others (Figure 7B).

In addition, pyroptosis is critical for the formation of the tumor‐immune microenvironment. We evaluated 176 DEGs in the two risk subgroups using the “limma” R package to better describe the immune‐related signatures of the two risk categories. CIBERSORT results showed that naive B cells, T cells, resting CD4 memory cells, resting natural killer (NK) cells, and resting monocytes and mast cells were significantly lower in the high‐risk subgroup than in the low‐risk subgroup. Regulatory T cells (Tregs), M0 macrophages, and resting dendritic cells (DCs) were significantly higher in the high‐risk subgroup than in the low‐risk subgroup (Figure 7C). We also compared immune cell infiltration and immune‐related pathway activity between the two risk subgroups using ssGSEA. Activated DCs, macrophages, and Tregs were enriched in the high‐risk subgroup, while B cells, mast cells, NK cells, and plasmacytoid DCs were enriched in the low‐risk subgroup (Figure 7D). Moreover, cytolytic activity, IFN response, and MHC class 1 were significantly different between the two groups (Figure 7E). In general, there was stronger immune activity in the low‐risk subgroup than in the high‐risk subgroup.

### 3.8 The association between *NLRP6* expression and clinicopathological variables

**FIGURE 8:**
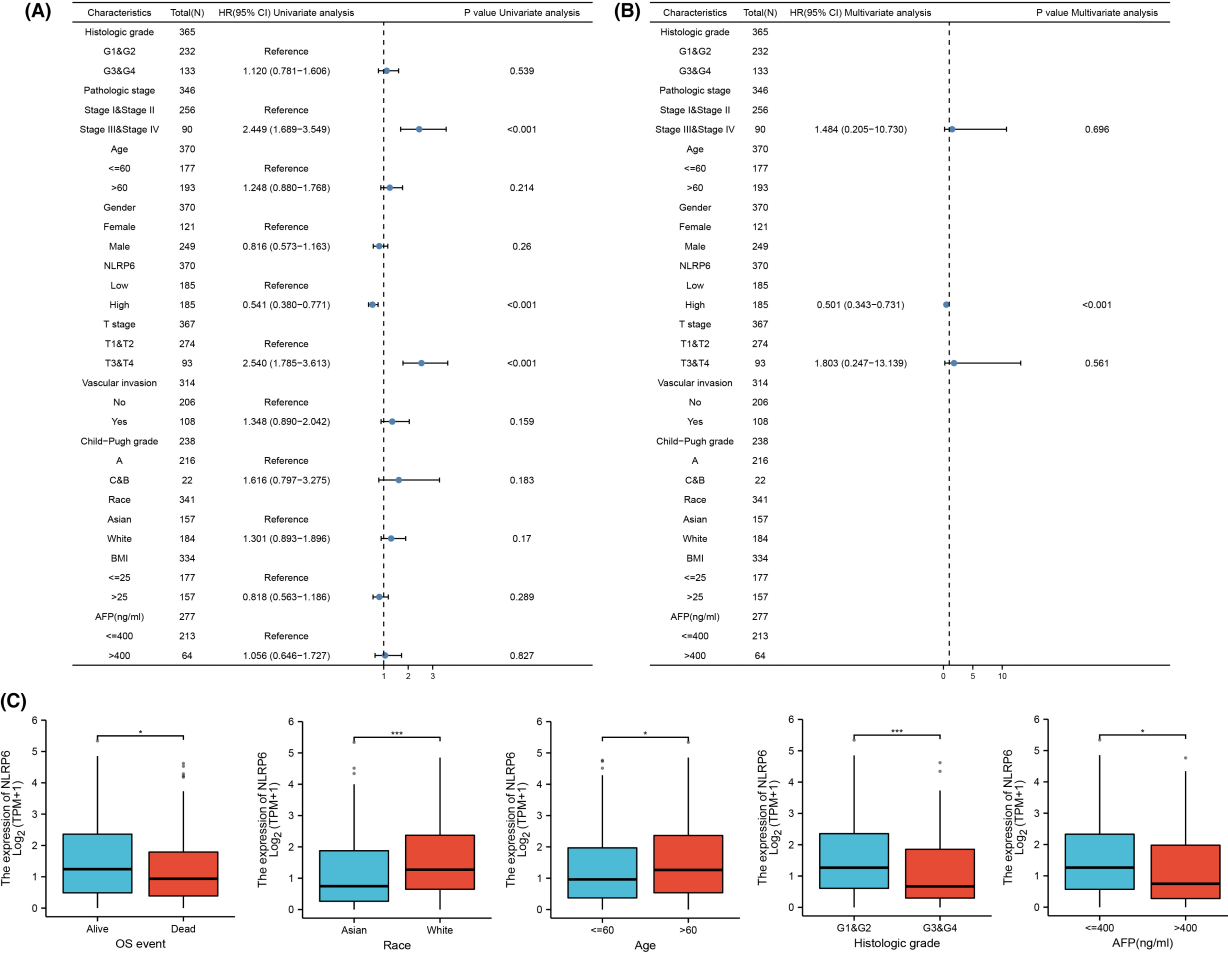
The association between *NLRP6* expression and clinicopathological parameters. (A) According to univariate analysis, tumor T stage, pathological stage, and *NLRP6* expression were correlated with prognosis. (B) According to multivariate analysis, *NLRP6* expression was an independent protective factor. (C) Association between *NLRP6* expression and clinicopathological parameters

The validation cohort also revealed that *NLRP6* is associated with better outcomes (Figure S2). As a result, we conclude that it is a tumor suppressor gene related to the pyroptosis signature. The relationship between *NLRP6* expression and clinical features of LIHC was investigated, and the findings revealed that *NLRP6* expression was significantly associated with age, race, tumor grade, AFP level, and OS event (Table 1). Univariate analysis showed that *NLRP6* expression, pathologic stage, and T stage were prognostic risk factors (Figure 8A). Because there were only four patients with lymph node metastasis and distant metastasis in TCGA‐LIHC, we did not discuss them here. *NLRP6* expression was found to be an independent predictive risk factor in multivariate analysis (Figure 8B). Furthermore, NLRP6 expression was significantly reduced in patients who had died, AFP > 400, Grade G3 + G4, Asian, and age ≤ 60 (Figure 8C).

**TABLE 1:** Correlation between NLRP6 expression level and clinicopathological parameters

Characteristic	Low expression of NLRP6	High expression of NLRP6	*p*
*n*	185	186	
Age, *n* (%)			0.049
≤60	98 (26.5%)	79 (21.4%)	
>60	86 (23.2%)	107 (28.9%)	
Gender, *n* (%)			0.395
Female	56 (15.1%)	65 (17.5%)	
Male	129 (34.8%)	121 (32.6%)	
Race, *n* (%)			0.019
Asian	92 (25.6%)	66 (18.4%)	
Black or African American	6 (1.7%)	11 (3.1%)	
White	82 (22.8%)	102 (28.4%)	
BMI, *n* (%)			0.054
≤25	97 (29%)	80 (23.9%)	
>25	69 (20.6%)	89 (26.6%)	
Histologic grade, *n* (%)			0.006
G1	20 (5.5%)	35 (9.6%)	
G2	81 (22.1%)	96 (26.2%)	
G3	72 (19.7%)	50 (13.7%)	
G4	9 (2.5%)	3 (0.8%)	
T stage, *n* (%)			0.546
T1	84 (22.8%)	97 (26.4%)	
T2	51 (13.9%)	43 (11.7%)	
T3	43 (11.7%)	37 (10.1%)	
T4	7 (1.9%)	6 (1.6%)	
N stage, *n* (%)			0.624
N0	131 (51.2%)	121 (47.3%)	
N1	3 (1.2%)	1 (0.4%)	
M stage, *n* (%)			0.355
M0	139 (51.5%)	127 (47%)	
M1	1 (0.4%)	3 (1.1%)	
Pathologic stage, *n* (%)			0.354
Stage I	79 (22.8%)	92 (26.5%)	
Stage II	49 (14.1%)	37 (10.7%)	
Stage III	45 (13%)	40 (11.5%)	
Stage IV	2 (0.6%)	3 (0.9%)	
AFP(ng/ml), *n* (%)			0.014
≤400	92 (33.1%)	121 (43.5%)	
>400	40 (14.4%)	25 (9%)	
Child‐Pugh grade, *n* (%)			0.495
A	104 (43.5%)	113 (47.3%)	
B	12 (5%)	9 (3.8%)	
C	0 (0%)	1 (0.4%)	
OS event, *n* (%)			0.003
Alive	106 (28.6%)	135 (36.4%)	
Dead	79 (21.3%)	51 (13.7%)	

*NLRP6* might negatively regulate HCC progression through targets of E2F and MYC and be associated with increased immune infiltration.

**FIGURE 9:**
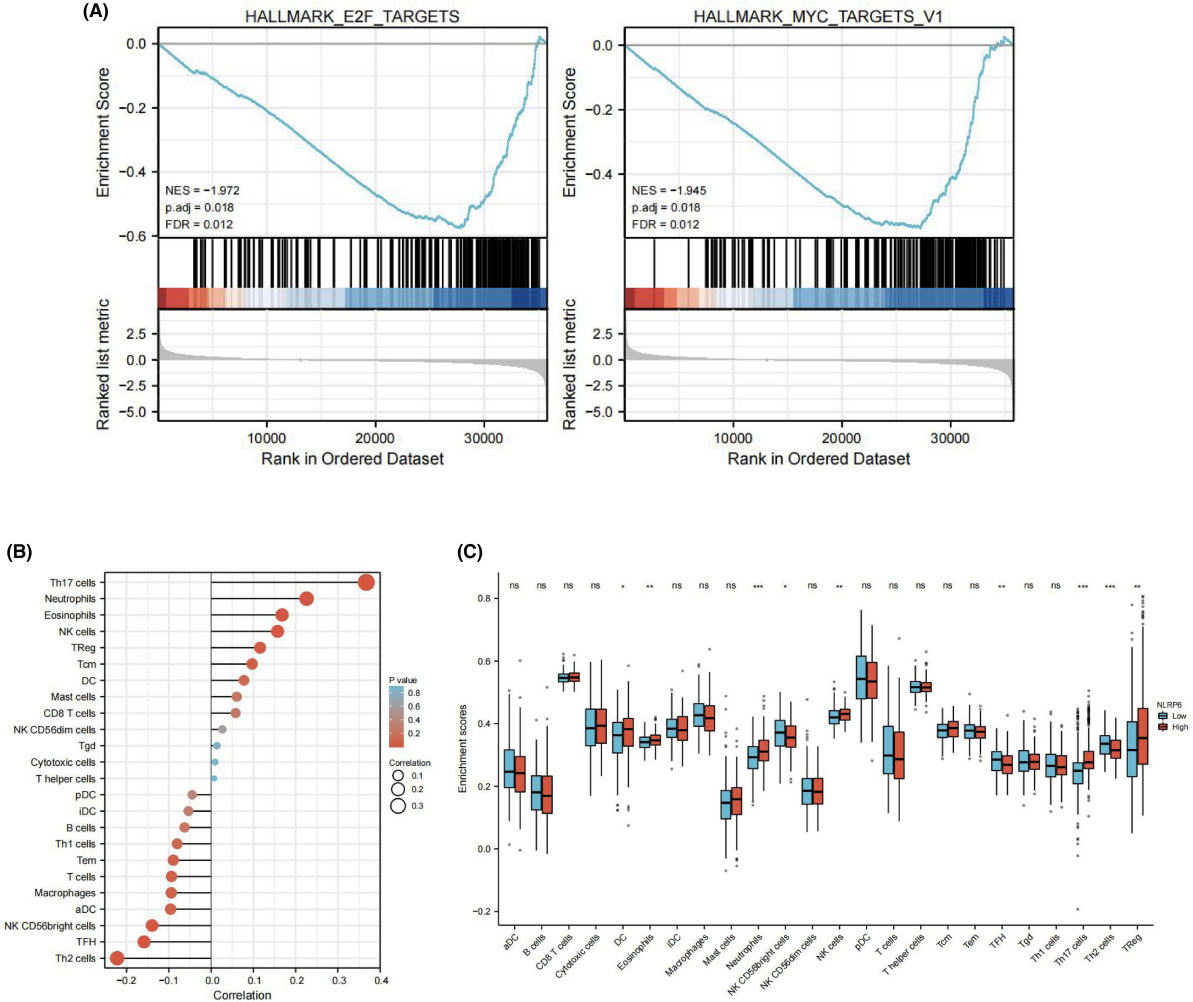
Functional enrichment analysis and immune infiltration analysis of *NLRP6*. (A) *NLRP6* was negatively correlated with E2F and MYC targets. (B) Association between *NLRP6* expression and immune cells. (C) Immune cell differences in HCC patients with high and low *NLRP6* expression

We applied GSEA on the basis of *NLRP6* expression to further understand the mechanism of HCC progression. We found eight negatively regulated pathways and four positively regulated pathways (Figure S3), and E2F and MYC targets were the top enriched gene signature (Figure 9A). E2F and MYC are associated with increased proliferation and aggressiveness in HCC; therefore, *NLRP6* may negatively regulate HCC progression through E2F and MYC targets. Overall, low *NLRP6* expression was correlated with increased aggressive behaviors in HCC.

Furthermore, we assessed differences in the relative proportions of multiple immune cells according to *NLRP6* expression (Figure 9B). The relative proportion of DC cells, eosinophils, neutrophils, NK cells, Th17 cells, and Tregs were all significantly and positively correlated with *NLRP6* expression, while the relative proportion of NK CD56 bright cells, follicular helper T cells (Tfh), and Th2 cells were significantly and negatively correlated with *NLRP6* expression (Figure 9C).

## 4 DISCUSSION

The incidence of HCC has been increasing, and its mortality rate is now ranked third among all malignancies, following only lung cancer and colorectal cancer.^1^, ^30^ Recently, significant attention has been paid to the development of new prognostic evaluation models on the basis of molecular typing to supplement the traditional pathological features and TNM staging. Pyroptosis has recently been identified as an innate immune response caused by multiple pathogens or non‐infectious factors that is mediated by caspases and inflammasome activity; furthermore, pyroptosis plays a complex role in tumor development. On the one hand, it can inhibit the development of malignant cells by inducing the expression of NLRP3 inflammasomes and activating the pyroptosis signaling pathway^31^; on the other hand, HCC is closely related to chronic inflammation and viral infection. During this process, pyroptosis leads to the release of intracellular pro‐inflammatory factors, which induce an inflammatory response that contributes to the development and progression of malignant tumors.^32^ Additionally, the induction of pyroptosis can improve the tumor immune microenvironment, and then activate the anti‐tumor immune response of T cells, thus improving the effect of immunotherapy.^33^ Therefore, it is important to understand the PRG signature in HCC, which can not only create a new prognostic model but also help to determine new therapeutic targets.

In this study, we first determined 26 pyroptosis‐related DEGs, among which six genes were related to prognosis, and C > G missense mutation was the most common mutation mode. Above all, we divided HCC patients into two clusters based on the different pyroptosis signatures, and we constructed a stable LASSO risk model to provide a new way of predicting the outcomes of HCC patients. We found that elevated levels of pyroptosis were associated with poor OS, suggesting that pyroptosis plays a significant role in the occurrence and development of HCC. Additionally, we propose that pyroptosis level and tumor grade interact dynamically, such that severe pyroptosis levels may promote to the evolution of HCC in a worse direction as it adapts to the inflammatory environment. Moreover, there were differences in functional enrichment analysis between the two risk subgroups. In short, low‐risk patients showed stronger immune activity.

*GMDSC, NOD2, SCAF11, GSDME, NLRP6,* and *CASP8* were used to construct risk models and were the most valuable DEGs in this study. *GSDME* determines the pattern of cell death. Specifically, when it is highly expressed, cytotoxic drugs induce tumor cell death through the caspase‐3‐dependent pyroptosis signaling pathway, and when its expression is low, the cell death pattern changes to apoptosis.^34^ According to previous reports, *GSDME* is generally expressed at low levels in tumor cells because of the hypermethylation of its promoter. In our study, *GSDME* was highly expressed in tumors and associated with poor prognosis. The same results were reported in a similar study published recently.^35^ Levels of *GSDME* methylation in HCC tumors samples were indeed increased. We suspect that this may be due to chronic inflammation or viral infection. However, the specific role of *GSDME* needs to be further explored. Among the Gasdermin family, *GSDMC* is less studied. Hou et al.^36^ found that *GSDMC*/caspase‐8 mediates a non‐canonical pyroptosis pathway that leads to more tumor necrosis, which was reported to contribute to tumor progression and increased resistance to chemotherapy and radiotherapy. Additionally, according to our findings, *GSDMC* not only correlates with poor OS but also with poor recurrence‐free survival. Multiple tumor‐related pyroptosis signatures have also reported that high *NOD2* and *SCAF11* expression are associated with poor prognosis, but unfortunately, their mechanism in tumors has not been uncovered.

*NLRP6* plays an important role in inflammation and host immune responses to intestinal microbiota.^37^ Additionally, *NLRP6* was a protective factor in the progression of non‐alcoholic fatty liver disease and obesity.^38^ Of the 33 pyroptosis genes we studied, *NLRP6* was the only one associated with better OS and DFS outcomes. The results suggest that *NLPR6* is a tumor suppressor gene that induces pyroptosis in HCC. Further analysis indicated that *NLRP6* expression was negatively associated with AFP > 400, Grade G3 + G4, Asian, and patient death. The primary etiologies of HCC in whites and Asians are different. In Asians, HCC is mainly due to chronic virus infection or diet, while in whites, HCC is primarily due to non‐alcoholic fatty liver disease.^39^ However, this seems contradictory because *NLRP6* is a protective factor for nonalcoholic fatty liver; thus, we assume that this is due to differences in genetic background. *NLRP6* is an independent risk factor, and AFP > 400, Grade G3 + G4, and death events reflected the poor prognosis. Finally, genome‐level changes seem to be a prerequisite for tumor evolution.

E2F is an important family of transcription factors that regulate the cell cycle, and both the E2F and MYC pathways can promote the progression of HCC.^40^, ^41^ Additionally, high *NLRP6* expression has been associated with more abundant neutrophils, NK cells, DC cells, and other tumor‐antagonizing immune cells. These results showed that *NLRP6* can be used as a prognostic biomarker for determining the prognosis and immune infiltration levels in HCC. The potential mechanism of the correlation between *NLRP6* and immune cells lies in that pyroptosis spreads danger signals from damaged or dead cells, mobilizing immune cells. Additionally, inflammasomes induce hyperactivation of DCs, which triggers enhanced T cell response and promotes T helper cells in the microenvironment to respond and secrete IL‐1β, driving the Th17 response.^9^, ^42^, ^43^

Two recent studies have also reported PRG signatures in HCC.^35^, ^44^ The risk model formulae of the three studies are different because of the different cohorts and PRG panels used. In particular, our risk models were constructed using larger cohorts. Although the genes involved in construction of the risk models were different, the AUC results for predicting prognosis were similar. Furthermore, we paid close attention to the pyroptosis genes related to prognosis and propose that *NLRP6* is a key protective gene of pyroptosis in HCC that has not been reported before. *NLRP6* may inhibit HCC progression by negatively regulating the E2F and MYC pathways and is associated with increased immune infiltration. On the basis of these results, we suggest that *NLRP6* plays an important role in HCC progression and could serve as a new prognostic marker and/or therapeutic target.

## 5 CONCLUSIONS

In conclusion, this study revealed the pyroptosis landscape of HCC and provided a promising clinical prognosis evaluation model. Moreover, we conclude that *NLRP6* can serve as a promising prognostic marker and a potential therapeutic target. These discoveries could provide new perspectives to understand the role of pyroptosis in HCC progression.

## AUTHOR CONTRIBUTIONS

Xin Gao (bioinformatics analysis and writing the manuscript).

Wen‐Xin Wang (bioinformatics analysis).

Xiao‐Lan Zhang (conceptualization and review).

## CONFLICT OF INTEREST

There are no potential conflicts of interest for the authors to disclose.

## Ethics approval and consent to participate

Not applicable.

## Consent for publication

Not applicable.

## Availability of data and materials

The corresponding author can provide the data and codes that support the conclusions of this study

## FUNDING

No specific funding has been received for this work.

## Supporting information


Appendix S1

Figure S1

Figure S2

Figure S3

## Data Availability

The datasets generated and/or analyzed during the current study are available at TCGA (https://portal.gdc.cancer.gov/) and GEO (https://www.ncbi.nlm.nih.gov/geo/) (GSE54236). The data and codes that support the findings of this study are available from the corresponding author.
