# Supplementary material for: A novel pyroptosis risk model composed of NLRP6 effectively predicts the prognosis of hepatocellular carcinoma patients
Source: Cancer Med. 2022 Jun 1;12(1):808–23. doi: 10.1002/cam4.4898 (PMC9844607; doi:10.1002/cam4.4898)
Supplement: Supplementary file 1 — Appendix S1 [file CAM4-12-808-s001.docx]

Table S1. Clinical characteristics between TCGA cohort and GEO cohort

| **Characteristic** | **TCGA cohort (n=374)** | **GEO cohort (n=78)** | **P value** |
| --- | --- | --- | --- |
| Gender, n (%) |  |  | 0.065 |
| Female | 122 (32.4%) | 17 (21.8%) |  |
| Male | 255 (67.6%) | 61 (78.2%) |  |
| Histologic grade, n (%) |  |  | 0.308 |
| Ⅰ+Ⅱ | 233 (62.3%) | 54 (69.2%) |  |
| Ⅲ+Ⅳ | 136 (36.4%) | 24 (30.8%) |  |
| NA | 5 (1.3%) | 0 (0%) |  |
| AFP (ng/ml), n (%) |  |  | 0.006 |
| <=400 | 215 (57.5%) | 71 (91.0%) |  |
| >400 | 65 (17.4%) | 7 (9.0%) |  |
| NA | 94 (25.1%) | 0 (0%) |  |
| OS event |  |  | ＜0.001 |
| Alive | 244 (65.2%) | 20 (25.6%) |  |
| Dead | 130 (34.8) | 58 (74.4%) |  |

NA, not available
