# Supplementary figures and images for: A novel pyroptosis risk model composed of NLRP6 effectively predicts the prognosis of hepatocellular carcinoma patients
Source: Cancer Med. 2022 Jun 1;12(1):808–23. doi: 10.1002/cam4.4898 (PMC9844607; doi:10.1002/cam4.4898)

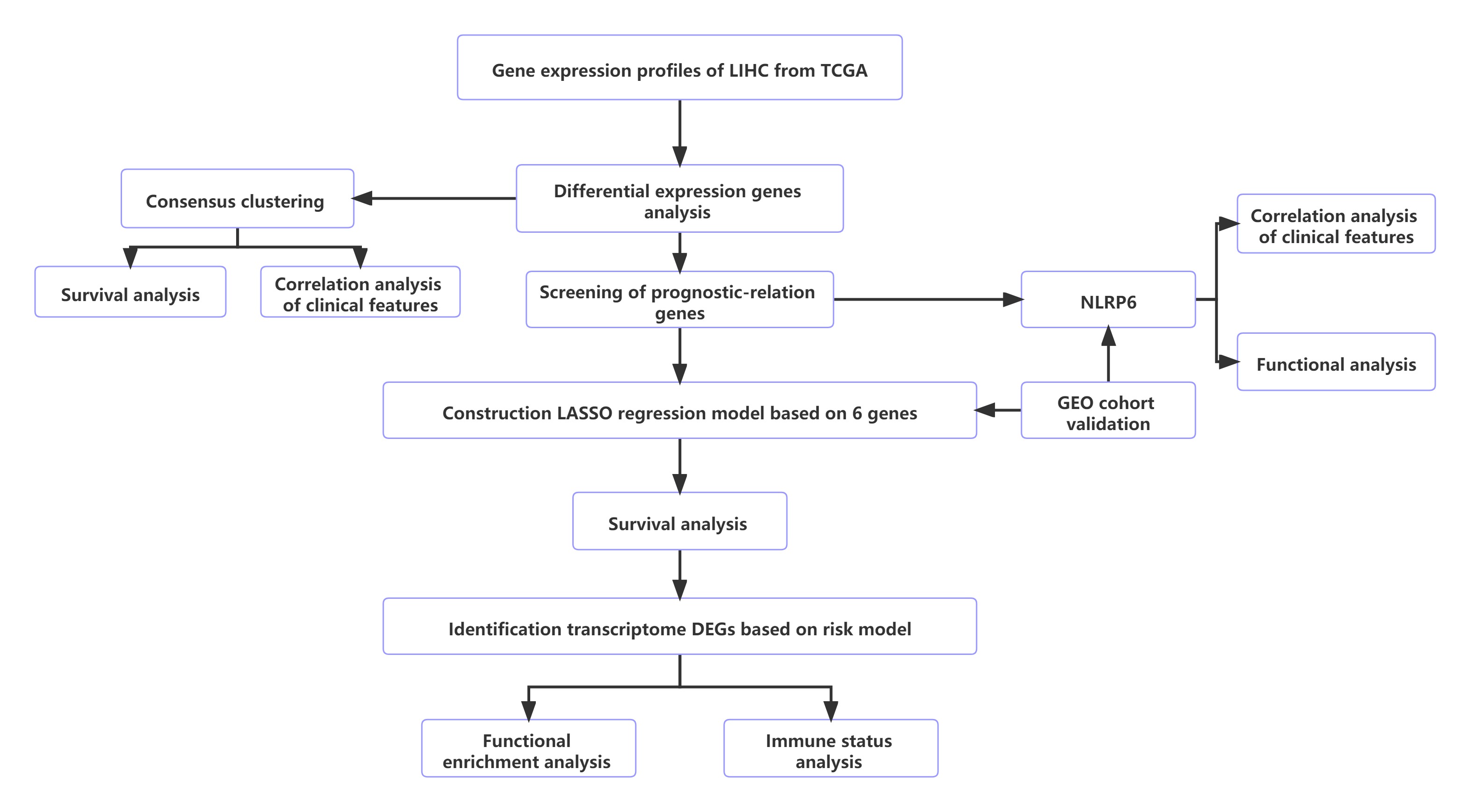

Supplement: Supplementary file 2 — Figure S1 [file CAM4-12-808-s002.jpg]

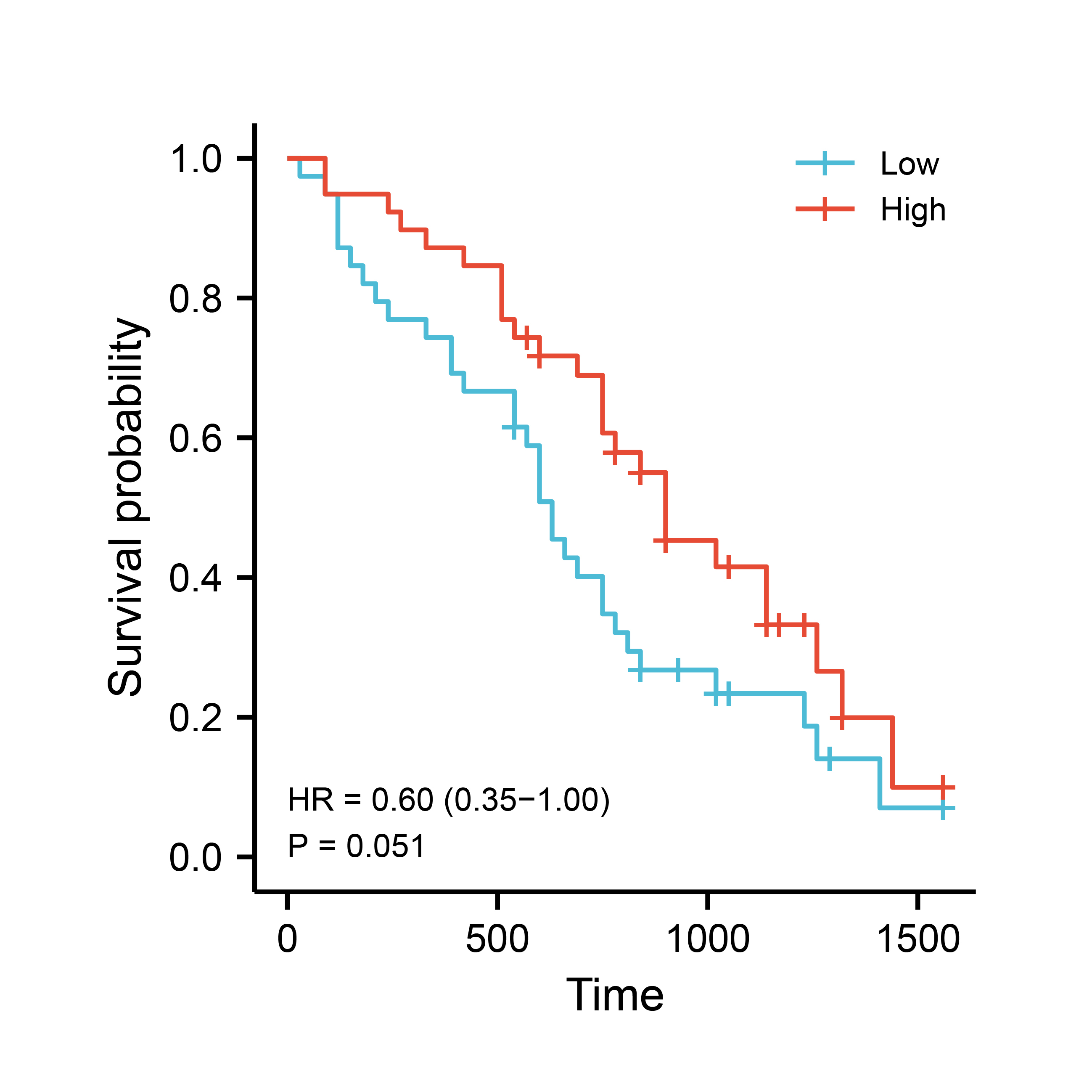

Supplement: Supplementary file 3 — Figure S2 [file CAM4-12-808-s004.jpg]

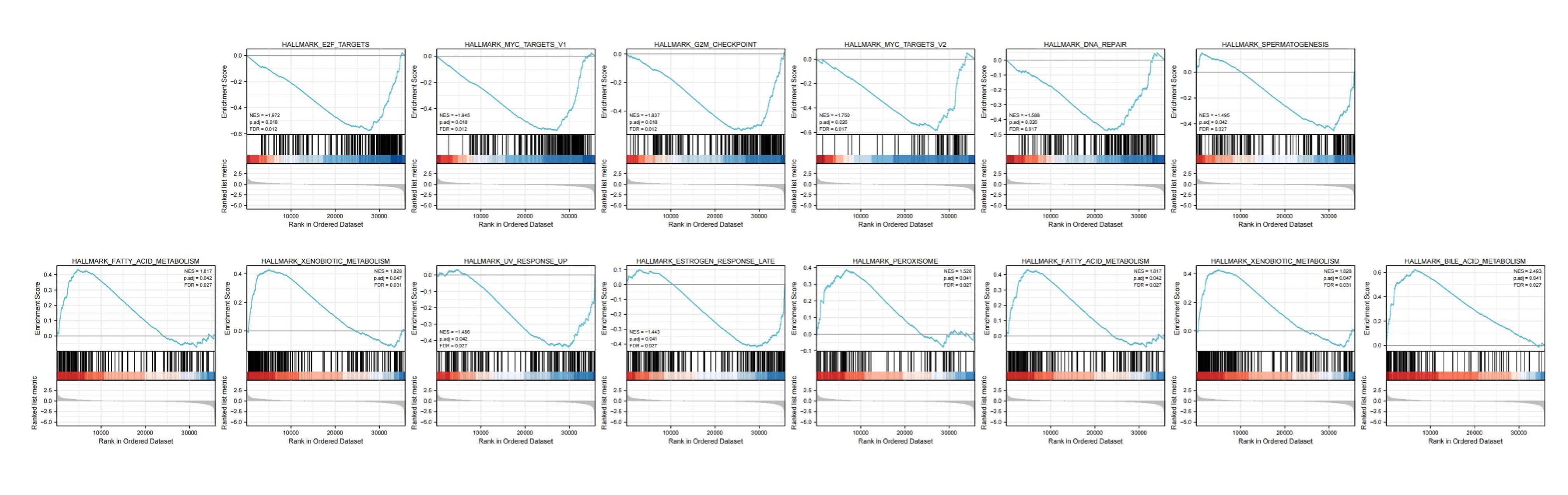

Supplement: Supplementary file 4 — Figure S3 [file CAM4-12-808-s003.jpg]
